# Description of a new species of *Stictochironomus* Kieffer, 1919 (Diptera, Chironomidae) from China based on DNA barcoding and adult morphology

**DOI:** 10.3897/zookeys.1276.180937

**Published:** 2026-04-03

**Authors:** Qi Lei, Wan-Ru Zhu, Zhi-Hang Yang, Xin-Yan Guo, Hai-Jun Tian, Xu-Sheng Guo, Rui-Lei Zhang, Jun-Ru Wang

**Affiliations:** 1 School of Fisheries, Xinyang Agriculture and Forestry University, Xinyang 464000, China Engineering Research Center of Environmental DNA and Ecological Water Health Assessment, Shanghai Ocean University Shanghai China; 2 Fishery Biological Engineering Technology Research Center of Henan Province, Xinyang 464000, China Fishery Biological Engineering Technology Research Center of Henan Province Xinyang China; 3 Engineering Research Center of Environmental DNA and Ecological Water Health Assessment, Shanghai Ocean University, Shanghai 201306, China School of Fisheries, Xinyang Agriculture and Forestry University Xinyang China

**Keywords:** China, DNA barcoding, morphology, new species, *
Stictochironomus
*

## Abstract

In April 2025, adult specimens of Diptera were collected in Henan Province, China., and based on an integrated analysis of DNA barcodes and morphology, a new species is formally described as *Stictochironomus longtanensis* Lei & Zhang, **sp. nov**. Adult males can be distinguished from all known congeners by a unique combination of morphological traits: a greater hypopygium ratio (1.98–2.11) and longer hypopygium (3.77–4.01), the presence of a white ring on the femur, the wing with a dark spot near the cross vein r-m, and a superior volsella bearing six bristles. Adult females are characterized by a small antennal ratio (AR 0.37–0.39); the wing with a dark spot near the cross vein r-m; genitalia with two ovoid, transparent seminal capsules; and a large, elliptical, densely setose cercus. The average interspecific genetic distance of the new species with congeners substantially exceeds the established threshold for species divergence in *Stictochironomus*, providing strong molecular evidence supporting this novel species. An updated taxonomic key to the known male adults of *Stictochironomus* from China is also provided.

## Introduction

*Stictochironomus* Kieffer, 1919 is one of the most widely distributed genera within the Chironomidae (Reistad 2023). To date, more than 30 species have been documented globally (Mukherjee et al. 2020; Konar 2021), with seven species confirmed in China (Song et al. 2024). Diagnostic morphological features, particularly pigmentation patterns on the wings and leg colouration, serve as key traits for species-level identification. For instance, *S. sticticus* (Fabricius, 1781) and *S. akizukii* (Tokunaga, 1940) exhibit a distinct dark spot near the r-m crossvein, whereas *S. juncaii* Qi, Shi & Wang, 2008 and *S. pictulus* (Meigen, 1830) display multiple coloured wing patches. Additionally, the number of setae on the superior volsella holds significant taxonomic value: *S. sticticus* bears six or more basal setae, in contrast to *S. akizukii*, which possesses only four (Qi et al. 2008). Consequently, integrating molecular approaches is essential for achieving a comprehensive understanding of species diversity and evolutionary dynamics within Chironomidae (Cranston et al. 2010). DNA barcoding, pioneered by Hebert et al. (2003), employs the mitochondrial cytochrome c oxidase subunit I (COI) gene as a standardized molecular marker. This method relies on the existence of a distinct “barcoding gap,” in which interspecific genetic divergence consistently exceeds intraspecific variation across most animal taxa. As a result, DNA barcoding has been widely applied within the Chironomidae for species identification, detection of cryptic species, and association of different life stages and sexes.

Lin et al. (2015) demonstrated that COI sequences could accurately delineate 94.6% of morphospecies within the genus *Tanytarsus* van der Wulp, 1874 based on an analysis of 2,790 DNA barcodes. A genus-specific genetic divergence threshold of 4–5% was established to define species boundaries. Song et al. (2018) achieved a 94.4% identification success rate across 3,670 barcodes in the highly diverse genus *Polypedilum* Kieffer, 1912, with phylogenetic analyses revealing multiple species complexes indicative of extensive cryptic diversity. The utility of DNA barcoding for life stage association was conclusively demonstrated by Lei et al. (2020), who successfully matched undescribed larvae and females of *Polypedilum bullum* Zhang & Wang, 2004 to conspecific males using COI sequences, with the maximum intraspecific divergence (3.96%) well below the genus-level threshold. Collectively, these studies confirm the robustness of DNA barcoding in addressing key challenges in chironomid systematics, including species delimitation, detection of cryptic diversity, and ontogenetic stage linkage.

In a recent systematic revision of the genus *Stictochironomus* from China, Song et al. (2024) employed COI barcode data to support the delineation of two species (*S. quadrimaculatus* Song & Qi, 2024 and *S. trifuscipes* Song & Qi, 2024). Furthermore, analysis of 704 genetic DNA sequences revealed remarkably high intraspecific divergence, reaching up to 16.81% in certain taxa such as *S. sticticus* and *S. pictulus*, which were resolved into multiple distinct lineages. This pattern strongly suggests the presence of cryptic species. Concurrently, ASAP analysis established a genetic threshold of 4.5–7.7%, providing an empirical benchmark for future species delineation within *Stictochironomus*.

In this study, male and female specimens of the Chironomidae collected from China were collected in the field and identified as a new species within the genus *Stictochironomus* based on DNA barcoding and morphological analyses. The species is described as *Stictochironomus longtanensis* sp. nov., named after its type locality, Longtan. Morphological characteristics of both sexes, along with COI barcode sequences, are provided, and key diagnostic features for the identification of adult males within this genus are presented.

## Materials and methods

### Sample collection

Adult specimens were collected using a sweep net in Longtan Village, Xinyang City, Henan Province, China (31.9343°N, 113.9264°E) on 4 April 2025. The specimens were immediately preserved with 85% ethanol and stored at −20 °C in the laboratory.

### Sample processing

DNA was extracted from thoracic muscle tissue and associated legs of individual adult specimens using the Ezup Column Animal Genomic DNA Purification Kit (Liu et al. 2023). All extractions were performed at the College of Fisheries, Xinyang Agriculture and Forestry University, Henan Province, China. Following DNA extraction, exoskeletons (including thorax, wings, legs, and antennae) were recovered and permanently mounted in Euparal on glass slides for morphological examination following the protocol of Sæther (1980). The holotype, paratypes, and mitogenomic DNA extracts of the new species are permanently deposited in the Aquatic Organism Specimen Laboratory, College of Fisheries, Xinyang Agriculture and Forestry University, Xinyang, China (hereinafter referred to by the institutional acronym **XYFU**).

The COI gene was amplified using the universal primers LCO1490 and HCO2198 (Folmer et al. 1994). The 25 μL PCR reaction mixture contained 12.5 μL of 2× Taq Master Mix, 0.5 μL of each primer (10 μM), 1 μL of template DNA, and 10.5 μL of ddH_2_O. Thermal cycling conditions were as follows: initial denaturation at 95 °C for 4 min; 40 cycles of denaturation at 94 °C for 45 s, annealing at 52 °C for 45 s, and extension at 72 °C for 1 min; followed by a final extension at 72 °C for 10 min. PCR products were verified on a 1.0% agarose gel and purified for bidirectional Sanger sequencing at Wuhan AuGCT Biotech Co., Ltd, China.

### Data analysis

High-quality sequences with clear chromatogram peaks were assembled and edited into contiguous sequences using SeqMan in Lasergene v. 7.0 (DNASTAR; Swindell and Plasterer 1997). The resulting consensus sequences were submitted to the Barcode of Life Data System (BOLD; Ratnasingham and Hebert 2007) where they were assigned GenBank accession numbers PV929838 to PV929841.

For comparative analysis, all publicly available COI sequences of the genus *Stictochironomus* were retrieved from BOLD (Table 1). The initial dataset was curated by removing duplicate and unidentified entries, resulting in a final dataset of 105 sequences designated as “DS-STCHLQ” (https://doi.org/10.5883/ds-stchlq), which was used for phylogenetic analysis.

**Table 1.** GenBank and BOLD accession numbers for sequences used in this study.

Species	GenBank/BOLD accession numbers
*Stenochironomus gibbus*	MZ656608, MZ657365
*Stictochironomus akizukii*	LC462294, LC462295
*Stictochironomus devinctus*	BBDIT1465-12, BBDIT1469-12, JF867700, JF867706, JF867730, JF867733, JN291594, KP954648KP954647, OPPFO1748-17, OPPMM666-17
*Stictochironomus maculipennis*	CHURC631-09, ATNA616-15, CHMNO322-15
*Stictochironomus pictulus*	MT534725, LC773102, MZ656195, BSCHI766-17, BSCHI767-17, LC462293, MZ661000, MZ659643, MZ657768, AB838702, AB838703, JCDB197-15, JCDB198-15, JCDB199-15
*Stictochironomus psilopterus*	HM406068, MT048162, HM406070, HM405917, HM405929, HM405981
*Stictochironomus rosenschoeldi*	HELAC812-21, CHRFI666-11, URESI026-18, JN265080, MZ660654, MZ632652, MZ631000, MZ658434
*Stictochironomus sinsauensis*	JF412167, JF412164, JF412165, JF412162, JF412163, JF412166
*Stictochironomus sticticus*	LC050944, MN667024, LC050941, JCDB282-15, MZ629576, MZ658667, LC773136, LC772993, LC772994, LC050943, FCHAR8795-19, MZ659009, MN677866, LC772991, LC772990, MIDGE946-11, LC772992, LC773297, MZ659999, JF870849, LC050945, JCDB281-15, MN683315, LC050942, URESI019-18, JCDB190-15, JCDB191-15, MN674299, MN665979
*Stictochironomus unguiculatus*	KR434480, JF877992, KR435150, KR442450, KR443825, HQ937665, HQ937794, HQ937646, HQ937651, HQ937661, HQ937679, HQ937680, HQ937697, HQ937717, HQ937728, HQ937744, JF874324, KR444733, KR429514, HQ937772, KR441360, KR435235

We aligned sequences using the MUSCLE algorithm in BioEdit v. 7.0 (Tippmann 2004). This alignment was analysed in MEGA v. 7.0 (Kumar et al. 2016) to construct a neighbour-joining phylogenetic tree under the Kimura 2-parameter (K2P) model, with *Stenochironomus gibbus* (Fabricius, 1794) designated as the outgroup and branch support assessed through 1,000 bootstrap replicates. Genetic distances were also calculated to quantify sequence divergence (Song et al. 2022).

## Results

### Stictochironomus longtanensis

Taxon classificationAnimaliaDipteraChironomidae

Lei & Zhang sp. nov.

95DC562F-3CEA-53D1-98EA-7BC992591C0B

https://zoobank.org/3E482358-7144-4229-AD12-35FCFAC334BD

Figs 1, 2, 3

#### Type material.

***Holotype***: • ♂ (BOLD ID: HHC011-25; Field ID: Chironomidae*Stictochironomus* S1), permanently deposited in XYFU (catalogue number: XYFU-HHC011); China, Henan Province, Xinyang City, Shihe District, Longtan Village, 31.9343°N, 113.9264°E, 4.IV.2025, leg. Q. Lei, sweep net. ***Paratypes***: • 2 ♂♂ (BOLD IDs: HHC012-25, HHC016-25), • 2 ♀♀ (BOLD ID: HHC013-25, HHC014-25), same data as holotype, same depository (catalogue numbers: XYFU-HHC012, XYFU-HHC013, XYFU-HHC014, XYFU-HHC016)

#### GenBank accession numbers.

PV929838–PV929841.

#### Etymology.

The specific epithet is derived from the name of type locality, Longtan village.

#### Diagnostic characteristics.

Adult males of this new species can be distinguished from other known *Stictochironomus* by the following combination of morphological characters: hypopygium ratio large (1.98–2.11; mean: 2.06) and hypopygium value large (3.77–4.01; mean: 3.91); abdomen and hypopygium brown; white ring present on each side of femur; wing with a dark spot near cross vein r-m; superior volsella with six basal setae. Adult females can be distinguished from other known *Stictochironomus* species by the following combination of characters: antennal ratio small (AR 0.37–0.39); wing with a dark spot near cross vein r-m; genitalia with two ovoid, transparent seminal capsules; and cercus large, elliptical, and densely setose.

#### Description.

**Adult male** (*n* = 3) (Table 2). Total length 6.77–7.46 mm, mean: 7.20 mm. Wing length 3.55–3.87 mm, mean: 3.77 mm. Total length/wing length 1.90–1.91 mm, mean: 1.91 mm. Wing length/length of profemur 2.22–2.45, mean: 2.36.

**Table 2.** Leg length (µm) and proportions (*n* = 3) of adult males of *Stictochironomus longtanensis* sp. nov.

Leg	fe	ti	ta1	ta2	ta3	ta4	ta5	LR
P1	1577–1627, mean: 1600	1394–1558, mean:1475	1662–1775, mean: 1718	926–1016, mean: 971	700–795, mean: 748	599–667, mean: 633	339–354, mean: 346	1.13–1.14, mean: 1.13
P2	1491–1756, mean: 1618	1420–1566, mean: 1513	813–891, mean: 860	480–517, mean: 503	342–394, mean: 372	217–267, mean: 249	129–177, mean: 159	0.56–0.57, mean: 0.57
P3	1865–2190, mean: 1990	1630–1793, mean: 1717	1167–1250, mean: 1195	674–764, mean: 724	489–592, mean: 543	300–348, mean: 324	174–227, mean: 205	0.68–0.70, mean: 0.70

***Colouration***. Head yellow and thorax brown. Legs distinctly annulated, with one white ring on femur and two white rings on tibia. On fore and mid legs (P1, P2), tarsomere I pale yellow at base and brown at apex, and tarsomeres II–V uniformly brown.

***Head***. AR 2.56–2.60, mean: 2.58. Temporal setae 15–19, mean: 18, including 4–5 (mean: 5) inner verticals; 5–8 (mean: 7) outer verticals; 6 postorbitals. Clypeus with 21–26 (mean: 24) setae. Tentorium 219–226 μm (mean: 223 μm) long, 69–94 μm (mean: 82 μm) wide. Palpomere lengths (in μm): 52–58 (mean: 55); 97–105 (mean: 101); 183–195 (mean: 189); 223–233 (mean: 228); 261–322 (mean: 292).

***Thorax***. Acrostichals 14–18 (mean: 16); dorsocentrals 12–14 (mean: 13); scutellars 27–28 (mean: 28); and prealars 7–9 (mean: 8).

**Figure 1.**
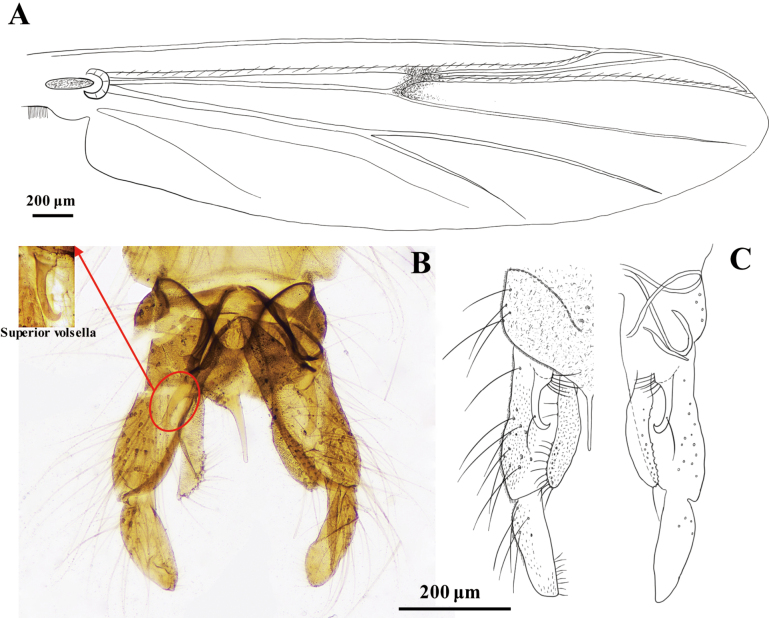
Adult male of *Stictochironomus longtanensis* sp. nov. **A**. Wing; **B, C**. Hypopygium.

***Wing*** (Fig. 1A). VR 0.93–0.97 (mean: 0.95). R with 24–27 (mean: 26); R_1_ with 17–19 (mean: 18); R_4+5_ with 22–24 (mean: 23) setae. Squama with 13–14 (mean: 14) setae.

**Figure 2.**
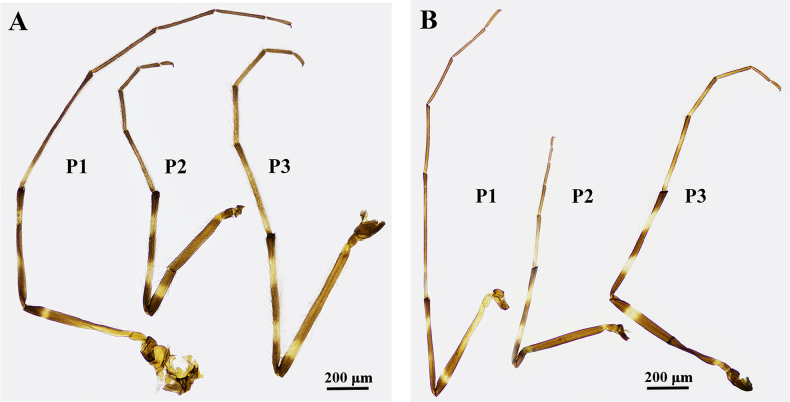
Legs of *Stictochironomus longtanensis* sp. nov. **A**. Adult male; **B**. Adult female.

***Legs*** (Fig. 2A). Tibial spur of mid leg 30–37 μm (mean: 34 μm) long; two tibial combs of mid leg 37–46 μm (41 μm) and 35–63 (46 μm) long, respectively. Tibial spur of hind leg 36–43 (mean: 40 μm) long; two tibial combs of hind leg 34–52 μm (mean: 40 μm) and 35–62 μm (mean: 49 μm) long, respectively. Tibial widths: fore leg, 68–76 μm (mean: 71 μm); mid leg, 82–94 μm (mean: 88 μm); hind leg, 86–105 μm (mean: 93 μm).

***Hypopygium* (Fig. 1B, C)**. Tergite IX with 7–9 (mean: 8) setae. Laterosternite IX with 8–11 (9) setae. Anal point slender, 108–135 μm (mean: 119 μm) long, with parallel lateral margins at the distal end and 8–13 (10) basal setae. Phallapodeme 91–94 (mean: 92 μm) long. Transverse stermapodeme 63–71 (mean: 66 μm) long. Superior volsella 106–114 μm (111 μm) long, with 6 basal setae and a long seta on distal 1/3. Inferior volsella 187–192 μm (mean: 189 μm) long with 11–15 (13) setae on distal end. Gonocoxite 335–418 μm (mean: 380 μm) long and 90–143 μm (112 μm) wide. Gonostylus stout, 169–198 μm (mean: 184 μm) long and 44–84 μm (62 μm) wide. HR 1.98–2.11 (mean: 2.06). HV 3.77–4.01 (mean: 3.91).

**Female** (*n* = 2) (Table 3). Total length 6.57–7.03 mm. Wing length 3.52–3.79 mm. Total length/wing length 1.85–1.87. Wing length/length of profemur 2.31–2.53.

**Table 3.** Length (µm) and proportions of (n = 2) of female adults of *Stictochironomus longtanensis* sp. nov.

Leg	fe	ti	ta1	ta2	ta3	ta4	ta5	LR
P1	1490–1500	1293–1406	1584–1592	829–910	642–703	567–596	272–301	1.13–1.23
P2	1534–1647	1372–1467	753–776	427–448	326–354	217–229	144–159	0.53–0.55
P3	1897–1930	1626–1644	1110–1186	644–649	516–518	311–323	183–184	0.68–0.72

***Colouration***. Head yellow; thorax brown; abdomen and hypopygium yellowish brown. Femur with one white ring; tibia with two white rings. Tarsomere I basally pale yellow, apically brown; tarsomeres II–V uniformly brown.

***Head***. AR 0.37–0.39. Temporal setae 16–20 including 4–6 inner verticals; 7–8 outer verticals; 5–6 postorbitals. Clypeus with 24–28 setae. Tentorium 206–207 μm long, 74–97 μm wide. Palpomere lengths (μm): 60-68; 97–107; 165–178; 222–227; 319–334.

**Figure 3.**
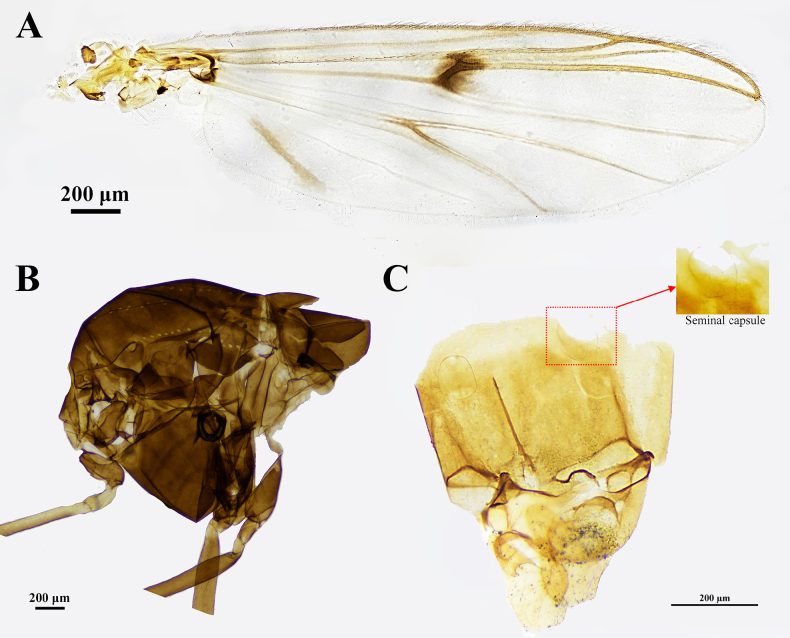
Adult female of *Stictochironomus longtanensis* sp. nov. **A**. Wing; **B**. Thorax; **C**. Hypopygium.

***Wing*** (Fig. 3A). VR 0.90–0.96. R with 30–37, R_1_ with 24–34, R_4+5_ with 51–56 setae. Squama with 15–21 setae.

***Thorax*** (Fig. 3B). Acrostichals 18–21, dorsocentrals 19, Scutellars 26–32, and prealars 6–8.

***Legs*** (Fig. 2B). Tibial spur of mid leg 36–41 μm long; two tibial combs 22–27 μm and 27–28 μm long, respectively. Tibial spur of hind leg 37–39 μm long; tibial combs of hind leg 29–31 μm and 34–39 μm long, respectively. Tibial widths: fore leg, 87–94 μm, mid leg, 102–116 μm, and the hind leg, 120–123 μm.

***Genitalia*** (Fig. 3C). Endophallus 228–237 μm long; basal inner process of ninth sternite 107–112 μm long. Notum 178–183 μm long. Cercus large, elliptical, densely setose, 229–243 μm long, 145–164 μm wide. Two seminal capsule vesicles, ovoid, and transparent.

#### Remarks.

The new species resembles *S. sticticus* and *S. akizukii* from China by sharing several characteristics, including the presence of rings on both the femur and tibia, a slender, elongated anal point with parallel lateral margins, and a hook-shaped superior volsella bearing a long seta at its proximal end. *Stictochironomus sticticus* may lack wing spots in some populations, although the specimens examined by Qi et al. (2008) possess a distinct dark spot near cross vein r-m. The inferior volsella is finger-like. Distinguishing features include that larger body size (6.77–7.46 mm in males and 6.57–7.03 mm in females) and a larger AR (2.56–2.60 in males and 0.37–0.39 in females). Notably, the hypopygium ratio (1.98–2.11, 2.06) and hypopygium value (3.77–4.01, 3.91) in male adults are greater. The female genitalia are characterized by having two ovoid, transparent seminal capsules and a large, elliptical, densely setose cercus measuring 229–243 μm long and 145–164 μm wide.

#### Distribution.

Currently, the new species is known from Henan and Zhejiang, China.

#### Analysis of DNA barcodes.

**Figure 4.**
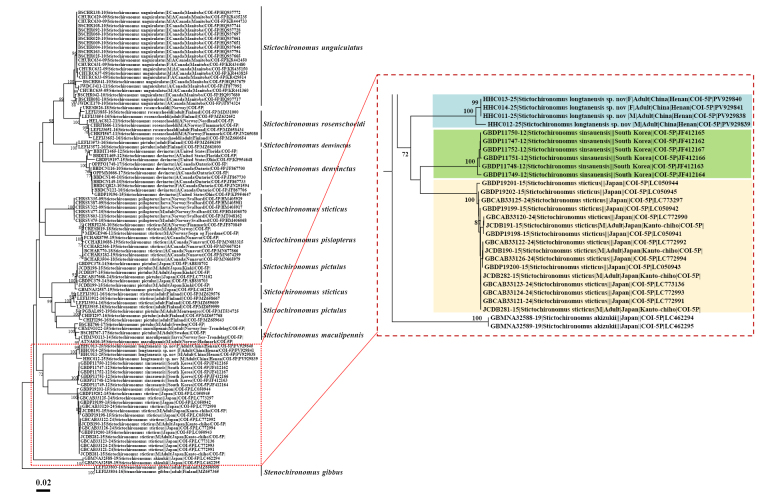
Neighbour-joining tree for species of *Stictochironomus* studied. Branch numbers indicate confidence levels derived from 1,000 bootstrap replicates, and the scale bar represents K2P genetic distance.

DNA barcoding successfully linked the morphologically distinct adult males and females of *Stictochironomus longtanensis* sp. nov. (Fig. 4). Pairwise genetic distance analyses (Table 4) revealed low intraspecific divergence, with a 0.00% genetic distance observed among three specimens (PV929840, PV929841, and PV929838) and only a 1.39% difference in the fourth specimen (PV929839). The average intraspecific genetic distance for *S. longtanensis* in this study was 0.69%, which falls within the typical range reported for conspecific individuals in the Chironomidae family.

**Table 4.** Pairwise genetic distances (%) among specimens of *Stictochironomus longtanensis* sp. nov.

Accession numbers	PV929838	PV929839	PV929840	PV929841
PV929838	0.00			
PV929839	1.39	0.00		
PV929840	0.00	1.39	0.00	
PV929841	0.00	1.39	0.00	0.00

The neighbour-joining (NJ) tree placed *S. longtanensis* in a clade with *S. sticticus* from Japan and *S. sinsauensis* from South Korea (Fig. 4). The average interspecific genetic distances between *S. longtanensis* and these congeners were 12.4% and 11.9%, respectively. Notably, these values substantially exceed the maximum intraspecific divergence threshold (4.5–7.7%) established for the genus *Stictochironomus* (Song et al. 2024), indicating the presence of a clear barcode gap. The minimum genetic distance from *S. longtanensis* to any recognized congener (11.40%) further supports its taxonomic distinctness (Suppl. material 1). Collectively, phylogenetic and genetic evidence strongly support the recognition of *S. longtanensis* as a new species.

##### An updated key to the known males of *Stictochironomus* from China

This key is primarily based on Song et al. (2024).

**Table:** 

1	Wing with a single dark spot around the cross vein r-m	**2**
–	Wing with multiple dark spots	**4**
2	Superior volsella with four basal setae; thorax yellow, with scutum, postnotum and scutellum brown (Sasa 1984)	***S. akizukii* (Tokunaga, 1940)**
–	Superior volsella with more than four basal setae; thorax and abdomen brown or yellowish brown	**3**
3	Superior volsella with six basal setae; gonostylus stout; AR ≥ 2.56; HV ≥ 3.77; legs distinctly annulated, each femur with a white ring and each tibia with two white rings	***S. longtanensis* sp. nov**.
–	Superior volsella with six or more basal setae; gonostylus slender and cylindraceous; AR < 2.56; HV < 3.77; fore femur with two brown rings; mid and hind femora each with a single apical pale-yellow ring, fore and mid tibiae with brown rings at both base and apex; hind tibia with two pale-yellow rings (Qi et al. 2008)	***S. sticticus* (Fabricius, 1781)**
4	Wing with smoky spots	**5**
–	Wing with four or more clearly separated spots	**6**
5	Inferior volsella with 14 setae; dorsocentrals 10–15; fore tibia predominantly black, with one subapical pale ring	***S. multannulatus* (Tokunaga, 1938)**
–	Inferior volsella with 18–20 setae; dorsocentrals 17–20; fore tibia predominantly pale, with anterior two-fifths brownish	***S. trifuscipes* (Song & Qi, 2024)**
6	Posterior area of wing without smoky spots	**7**
–	Posterior area of wing somewhat smoky, with five spots; dorsocentrals 18; tibiae with three dark and two pale rings (Qi et al. 2008)	***S. juncaii* (Qi, Shi & Wang, 2008)**
7	Wing with six spots; dorsocentrals 7; inferior volsella with 12 setae; fore femur pale with three dark rings, tibia with four dark rings	***S. quadrimaculatus* (Song & Qi, 2024)**
–	Wing with four spots; dorsocentrals 18–22; inferior volsella with 20–23 setae; fore femur largely brown, with a short preapical pale ring, tibia with three dark rings (Sasa and Suzuki 1998)	***S. pictulus* (Meigen, 1830)**

## Supplementary Material

XML Treatment for Stictochironomus longtanensis

## Additional information

### Use of AI

No artificial intelligence tools were used in this study.

### Author ORCIDs

Qi Lei https://orcid.org/0000-0002-3842-2394

Rui-Lei Zhang https://orcid.org/0000-0001-5953-3192

### Data availability

All of the data that support the findings of this study are available in the main text or Supplementary Information.

## Supplementary materials

Supplementary material 1 Pairwise genetic distances Data type xlsx Explanation note **appendix S1**. The Pairwise genetic distances (%) among specimens of *Stictochironomus* in this study. https://binary.pensoft.net/file/1579205This dataset is made available under the Open Database License (http://opendatacommons.org/licenses/odbl/1.0/). The Open Database License (ODbL) is a license agreement intended to allow users to freely share, modify, and use this Dataset while maintaining this same freedom for others, provided that the original source and author(s) are credited. Qi Lei, Wan-Ru Zhu, Zhi-Hang Yang, Xin-Yan Guo, Hai-Jun Tian, Xu-Sheng Guo, Rui-Lei Zhang, Jun-Ru Wang
